# Increasing flash droughts over China during the recent global warming hiatus

**DOI:** 10.1038/srep30571

**Published:** 2016-08-11

**Authors:** Linying Wang, Xing Yuan, Zhenghui Xie, Peili Wu, Yaohui Li

**Affiliations:** 1 RCE-TEA, Institute of Atmospheric Physics, Chinese Academy of Sciences, Beijing 100029, China; 2 University of Chinese Academy of Sciences, Beijing 100049, China; 3 LASG, Institute of Atmospheric Physics, Chinese Academy of Sciences, Beijing 100029, China; 4 Met Office Hadley Centre, Exeter EX1 3PB, UK; 5 Institute of Arid Meteorology, China Meteorological Administration, Lanzhou 730020, China

## Abstract

The recent global warming slowdown or hiatus after the big El Niño event in 1997/98 raises the questions of whether terrestrial hydrological cycle is being decelerated and how do the hydrological extremes respond to the hiatus. However, the rapidly developing drought events that are termed as “flash droughts” accompanied by extreme heat, low soil moisture and high evapotranspiration (ET), occurred frequently around the world, and caused devastating impacts on crop yields and water supply. Here, we investigate the long-term trend and variability of flash droughts over China. Flash droughts are most likely to occur over humid and semi-humid regions, such as southern and northeastern China. Flash drought averaged over China increased by 109% from 1979 to 2010, and the increase was mainly due to a long term warming of temperature (50%), followed by the contributions from decreasing soil moisture and increasing ET. There was a slight drop in temperature after 1997, but the increasing trend of flash droughts was tripled. Further results indicate that the decreasing temperature was compensated by the accelerated drying trends of soil moisture and enhanced ET, leading to an acceleration of flash droughts during the warming hiatus. The anthropogenic warming in the next few decades may exacerbate future flash drought conditions in China.

Drought is usually a creeping phenomenon that persists for several months or years, and affects the food and water security significantly^1^,^2^, and heatwave usually lasts for a few days with sustained high temperature and notable impacts on human mortality and environment^3^. The two types of extremes have quite different time scales, but now they have a higher concurrent probability in many regions across the world under global warming^4^, where increasing heatwave exacerbates drought condition, and drought in turn creates a favorable condition for heatwave^5^,^6^,^7^,^8^. The concurrent drought and heatwave events with low soil moisture and high evapotranspiration (ET) are recently termed as “flash drought”^9^,^10^ due to their rapid onsets, unusual intensity and devastating impacts^11^,^12^. For example, a moderate drought condition combined with a heatwave initiated a flash drought event over the central United States in May and early June 2012^9^, which was unexpected and brought a grand challenge for early warning due to limited prior external signals^13^,^14^, and led to widespread crop failure and billions of economic losses. Other flash drought events were reported in China^10^ and Europe. However, to date, there are limited studies that detect and attribute the change of flash droughts specifically.

Similar to traditional droughts, a change in flash droughts is not only associated with the change in terrestrial hydrological cycle, but also influenced by the change in terrestrial energy cycle (and the related heatwaves) as well as the interaction between the land and atmosphere^15^. Under global warming, the risk of heatwave is increasing^5^, and the terrestrial hydrological cycle is also expected to be intensified with increasing water vapor feedback^16^, leading to the changes in precipitation, soil moisture and ET, as well as characteristics of climate extremes (e.g., droughts). However, global mean temperature remained flat during the first decade of the twenty-first century^17^,^18^. The potential causes may include internal climate variability, such as the La Niña-like decadal cooling prevailed over the tropical Pacific^19^,^20^,^21^ and reduced radiative forcing due to the increased stratospheric aerosols concentrations^22^,^23^ together with the minimum solar activity around 2009^24^,^25^. During the warming hiatus, the trend for global land ET declined as well, although it was mainly associated with limited moisture supply^26^. Land ET is a crucial variable that affects the forming and evolution of flash droughts because the increase in ET can amplify the soil moisture anomalies and intensify the drought condition^9^,^27^. The soil moisture is also an important index for the identification of flash droughts, but the change in soil moisture is unclear^1^,^15^ due to the observation and model uncertainties. Given the slowdown of temperature and ET, and the uncertain change in soil moisture, it remains unclear what changes have occurred in the flash droughts and whether the hiatus is responsible for the changes.

In this study, we focus on flash droughts over China, which is one of the most sensitive areas to the climate change, suffering from warming and drying significantly^28^. Surface air temperature measurements for the period of 1979–2010 from over two thousand meteorological stations, and soil moisture and ET estimations from three global reanalysis products are used to investigate multidecadal changes in flash droughts over China and the underlying causes.

## Results

### Frequency of occurrence for flash droughts

**Figure 1:**
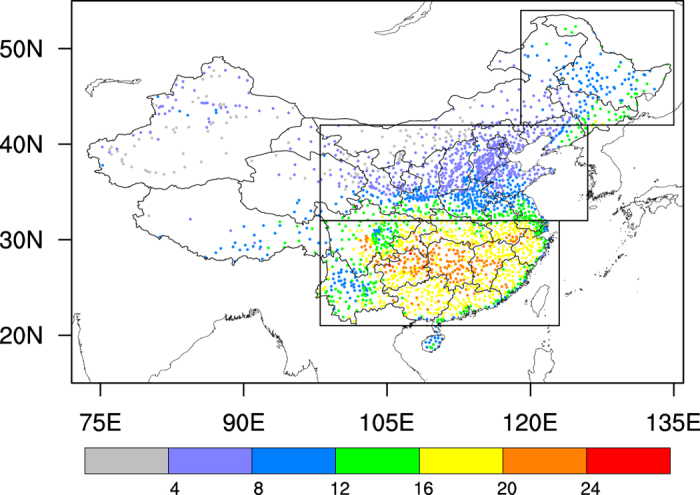
Ensemble mean frequency of flash drought events during the growing seasons (April to September). Flash drought pentad is defined as the pentad-mean surface air temperature (T) anomaly >one standard deviation, ET anomaly >0, and soil moisture percentile (SM%)<40%. If two or more consecutive flash drought pentads happen one after another, they will be treated as a single drought event. The ensemble mean is the average of the frequency from each reanalysis product. The unit is in events per decade. The boxes represent the locations of three sub-regions: southern China (21–32°N, 98–123°E), northern China (32–42°N, 98–126°E) and northeastern China (42–54°N, 119–135°E). The figure was created by the NCAR Command Language (Version 6.3.0) [Software]. (2016). Boulder, Colorado: UCAR/NCAR/CISL/TDD. http://dx.doi.org/10.5065/D6WD3XH5.

Here we use the pentad-mean conditions to capture the short duration of flash droughts during crop growing seasons when they mostly occur^29^. For each meteorological station and each pentad, a flash drought is defined as the condition in which surface air temperature anomaly is larger than one standard deviation, ET anomaly is positive, and the soil moisture percentile is lower than 40%^9^. The ensemble mean frequency of occurrence for flash droughts over the period of 1979–2010 is spatially very variable across the country owing to the influence of climate, soil and vegetation (Fig. 1). Flash droughts occur mostly in southern China with a mean frequency of 16–24 events per decade, followed by the northeastern China. In contrast, there are fewer flash drought events over the arid (northwestern China), semiarid and semi-humid (northern China) regions where vegetation is relatively sparse. This is easily understandable because over humid and semi-humid regions (e.g., southern and northeastern China), ET is mostly limited by energy supply as moisture is usually sufficient (even under a moderate drought condition). Positive ET anomalies, therefore, tend to occur concurrently with high temperature, creating a perfect condition for flash droughts. But for the water limited regions (e.g., northern and northwestern China), ET is very sensitive to soil moisture variations and a moderate dry condition may result in a significant reduction in the ET (i.e. negative ET anomalies), reducing the probability of the occurrence of flash droughts according to the definition. Another reason for the spatial distribution is the vegetation distribution, where a densely vegetated region will pump more water from deep soil during drought, leading to an increase in ET and consequently the occurrence of flash droughts. Therefore, flash droughts in China are most likely to occur over humid and semi-humid regions with dense vegetation. Replacing soil moisture anomalies with precipitation anomalies in the above calculation, one finds similar spatial distribution of frequency changes but with higher amplitudes (see Methods for details). On average, the mean duration of flash drought events is about 1.2 pentads (6 days) and the mean duration for each station varies between one to two pentads (5–10 days, Fig. S4).

### Trend and variability of flash droughts

**Figure 2:**
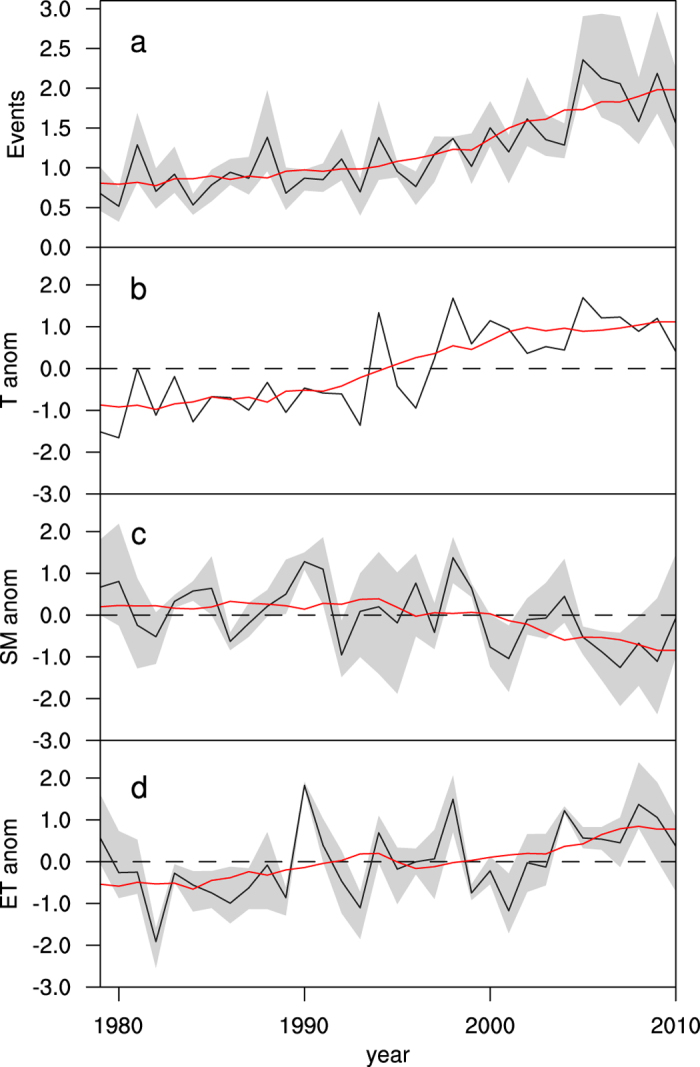
Interannual and decadal variations of ensemble mean flash drought event and its component variables averaged over China. (**a**) The number of flash drought events per year, (**b**) surface air temperature (T) anomaly, (**c**) soil moisture (SM) anomaly, (**d**) ET anomaly (black curves). The red curves are the 10-year running means and the grey shadows are the ranges of results from different reanalysis products. Except for the flash drought events (**a**) that are averaged from three reanalysis products directly, the component variables (**b–d**) are standardized for each reanalysis product before being averaged to facilitate the comparison. All statistics are based on those during the growing seasons.

**Figure 3:**
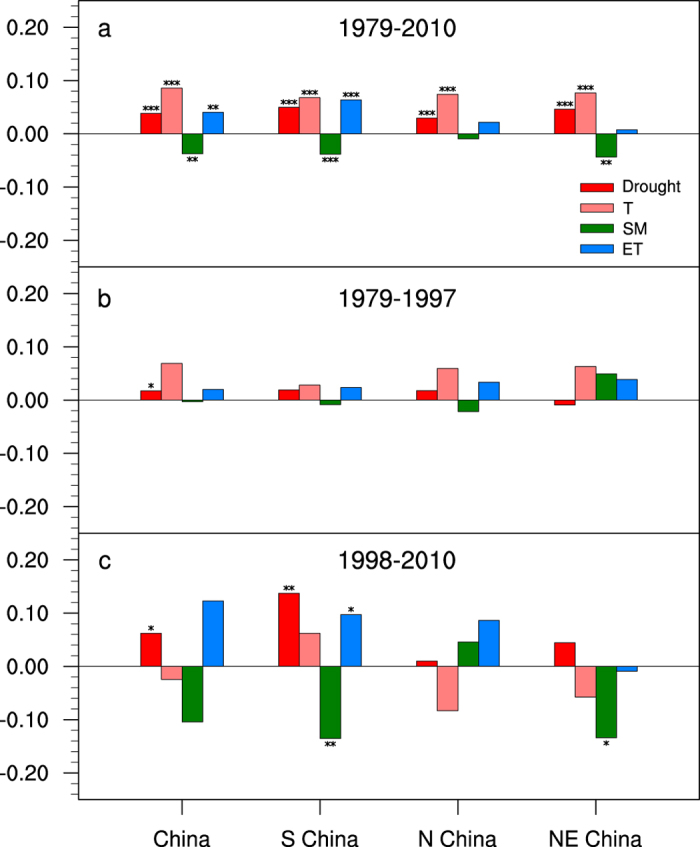
Mann-Kendall trends of flash drought event and its component variables of temperature (T), soil moisture (SM) and ET averaged over China and the sub-regions for the periods of 1979–2010 (**a**), 1979–1997 (**b**), and 1998–2010 (**c**), respectively (see Methods for the trend analysis). The sub-regions are defined as boxes in Fig. 1. Except for the flash drought events (**a**) that are averaged from three reanalysis products directly, the regional mean component variables (**b–d**) are standardized for each reanalysis product before being averaged to facilitate the comparison. The trends are calculated based on the ensemble mean variables from three reanalysis products. ***, **, and *, are the significances of the trends at the 99%, 95%, 90% confidence intervals respectively.

Figure 2a shows the number of flash drought events per year averaged over China during 1979–2010. For the whole period, despite some differences among three reanalysis products, they all indicate an increasing trend over China. The flash drought events increased by 109% from 1979 to 2010 with a statistical significance of p < 0.01 (see Methods for details). Similar trends (p < 0.01) can also be found in the sub-regions, with the highest increasing trend over southern China, followed by northeastern China, and northern China the lowest (Fig. 3a).

During the period of 1979–2010, the warming rate is 0.36 °C per decade (p < 0.01) over China, with a sharp increase in the 1990s and a stabilization in the 2000s (Fig. 2b). In addition, there is a decreasing trend for soil moisture (p < 0.05) and an increasing trend for ET (p < 0.05), although less significant than the change in temperature (Fig. 2c–d, Table S1). For three sub-regions, the trends of three component variables are similar to those for the entire China, but with less significance for the soil moisture and ET over northern and northeastern China (Fig. 3a, Table S1).

A multiple linear regression is applied to quantify the contributions of temperature, soil moisture and ET to the trend in flash drought (see Methods for details), and the regression explains over 81% of the variability of the flash droughts across China and its sub-regions. For the entire continental China, the contribution of the surface air temperature variability to the flash drought variability is the biggest (50.1%), followed by soil moisture (29.6%) and ET (20.3%). For the southern China, the flash drought variability is also mainly contributed by surface air temperature (48.5%), followed by soil moisture (37.7%) and ET (13.8%). The contributions of surface air temperature to the flash droughts over northern and northeastern China are again dominant (64.6% and 53.3%), and the secondary contributors are the ET (22.7%) and soil moisture (40.7%) respectively.

### Changes in the flash drought trend during the warming hiatus

From the analysis above, it is found that the surface air temperature variability accounts for most of the variability for the flash droughts across China and its sub-regions during the whole period of 1979–2010 (Fig. 3a). Before the big El Niño event in 1997/98, there was a warming trend over China, with a rate of 0.20 °C per decade (Fig. 3b). It switched to an opposite cooling trend with a rate of −0.05 °C per decade since 1998 (Fig. 3c), indicating that there is a warming hiatus in China. However, the increase in flash droughts does not seem to cease. The increasing trend over China for the period of 1998–2010 is 259% higher than for the period of 1979–1997 (Fig. 3b–c), and the difference in trends is statically significant (p < 0.1).

**Figure 4:**
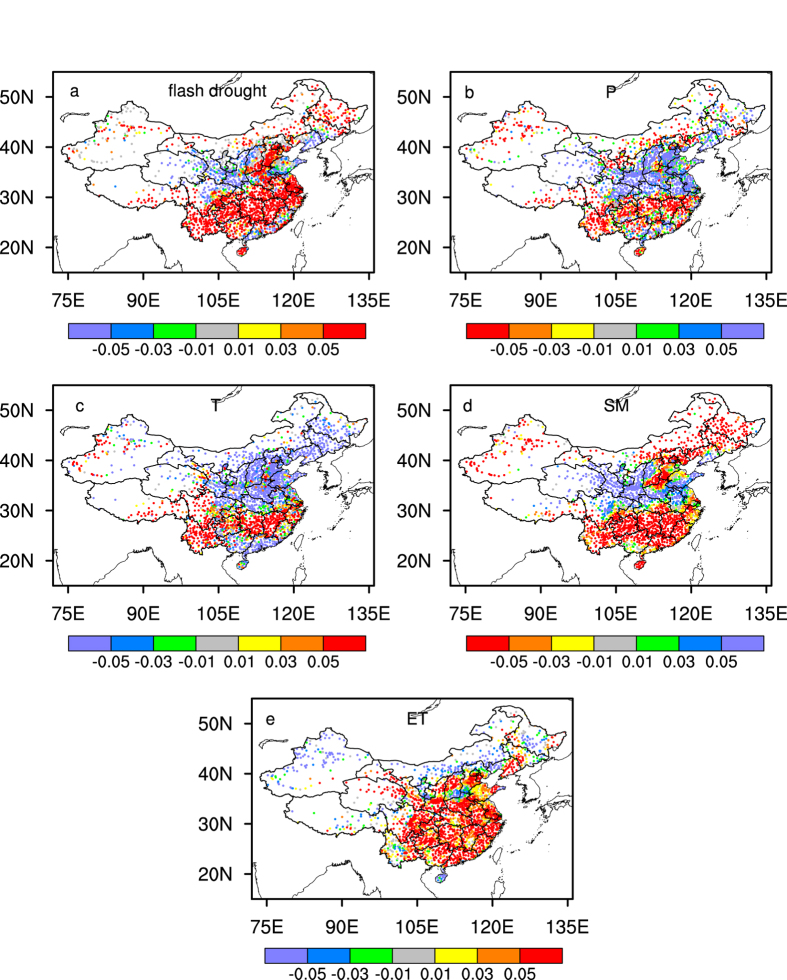
Ensemble means of the changes in trends between 1979–1997 and 1998–2010 for the flash droughts and different hydrometeorological variables. (**a**) Changes in trends for the number of flash drought events during April-September (pentads per year). (**b**) As for **a**, but for the trend changes for the standardized precipitation (P). (**c**) As for **b**, but for the standardized surface air temperature (T). (**d**) As for **b**, but for the standardized soil moisture (SM) anomalies. (**e**) As for **b**, but for the standardized ET. The figure was created by the NCAR Command Language (Version 6.3.0) [Software]. (2016). Boulder, Colorado: UCAR/NCAR/CISL/TDD. http://dx.doi.org/10.5065/D6WD3XH5.

In fact, the flash drought exhibits an accelerated increasing trend over most parts of China, especially in southern and northeastern China (Fig. 4a), although there is a warming hiatus across continental China except for the southwest and the Yangtze River basin (Fig. 4c). For the southern China, the significant increasing trend in flash drought (p < 0.05) in the last decade is mainly associated with an accelerated warming, an increased drying trend in soil moisture, and an accelerated increasing trend in ET (Figs 3c and 4d–e). But the increasing trend in temperature over southern China is not significant during 1998–2010 (Fig. 3c, Table S2) due to a decrease-increase-decrease triple pattern for the trend changes (Fig. 4c). For the northern China, the changes in flash drought trend are mixed (Fig. 4a), resulting in a decelerated increasing trend on average which is mainly caused by a cooling trend and a wetter soil condition (Figs 3c and 4c–d). For the northeastern China, the trend in flash drought is accelerated, which is mainly associated with the significant decrease of soil moisture (Fig. 4a,d).

## Discussion

Flash drought is a kind of agricultural drought in nature^9^, with concurrent events of extreme heat, soil water deficit and high vegetation transpiration demand. They are most likely to occur over humid and semi-humid regions (e.g., southern and northeastern China). This study uniquely investigates the trend and variability of the flash droughts over China, and is targeted at attributing the changes of flash droughts within the context of the understanding of the terrestrial water cycle in a changing climate.

Our results show that there are increasing trends for flash droughts over different regions in China, which is mainly associated with the temperature increase. This is similar to previous studies over California, where the global warming can increase the chance of concurrent droughts and heatwaves^30^,^31^. In fact, high temperature may substantially exacerbate the drought condition. For example, the 1977 drought in California was only a 50 year extreme event, while the 2014 drought that co-occurred with heatwave was a 200 year extreme event^30^.

In this study, the increasing trends of flash droughts do not decline after the big El Niño event in 1997/98. Although it is worthy of mentioning here that the trend statistics over 1998–2010 may not be robust due to the relative short period, the analysis does suggest that there exists significant increasing trends (p < 0.1) in flash droughts over China during the warming hiatus. The underlying mechanisms are attributed by investigating the changes in temperature, soil moisture and ET. We find that increasing flash droughts during the warming hiatus is mainly associated with the accelerated drying trend of soil moisture over humid and semi-humid regions, and the accelerated increasing trend of ET over low and middle latitude regions (Fig. 4). The accelerated drying trend of soil moisture over southern China is mainly associated with an accelerated decreasing trend in precipitation (Fig. 4b) that might be caused by decadal climate variability and changes in anthropogenic aerosols^32^. The accelerated increasing trend of ET over southern China seems to contradict with the drying trends both in precipitation and soil moisture, but it is actually coincident with previous findings that both the potential and actual ET exhibit increasing trends over southern China^26^. This might be due to an increasing atmospheric demand under drought conditions, where the change in ET over humid regions is not necessarily correlated with the change in soil moisture but rather with available energy, and less precipitation usually means more solar radiation. In addition, a greening trend^33^ with better vegetation conditions might also be responsible for the increasing ET.

The latest IPCC report suggests that global warming is going to continue well into the 21^st^ century. The observed increasing flash droughts reported in this study could potentially be strengthened further in the next few decades with more severe impacts on the water availability, agriculture and ecosystem. As flash droughts occur mostly in humid and semi-humid regions where people are less aware and less prepared, their impacts on human society and livelihood are much more severe. Different from traditional drought events, this new phenomenon demands urgent research into its mechanisms, monitoring and early warning system development.

## Methods

### Observational datasets

We used the daily surface air temperature and precipitation observations from 2474 China Meteorological Administration (CMA) meteorological stations during the period of 1961–2014. A quantile mapping method^34^ that matches the cumulative distribution functions (CDFs) from the station data and the Global Land Data Assimilation System version 2 (GLDAS-2)^35^ global gridded forcing data at a 0.25-degree resolution, was used to fill the missing surface air temperature station data. After this procedure, about 22 stations were excluded due to the scale mismatch over the coastal areas. Observed precipitation for the remained 2452 stations was also filled in the same manner. If the missing data remained after the quantile-mapping, an inverse distance weighting interpolation from the 0.25-degree gridded data was used to fill them.

### Reanalysis datasets

We used the soil moisture and ET from three global reanalysis products: GLDAS-2 3-hourly products at a 0.25-degree resolution, ERA-Interim^36^ 3-hourly products at a 0.25-degree resolution and NCEP Climate Forecast System Reanalysis (CFSR)^37^ 6-hourly products at a 0.3125-degree resolution. GLADS-2 provides the estimations of soil moisture and ET from a continuous simulation with the NOAH land surface model forced by meteorological observations. ERA-Interim and CFSR provide the estimations of land surface conditions by initializing a climate model every few hours. GLDAS-2 and ERA-Interim were verified against *in-situ* soil moisture observations over China, and showed some confidence in the drought analysis^10^. These datasets cover the base period from 1979 to 2010. First, we computed the daily mean of the variable of interest. Then, we matched the grid data to the corresponding or nearest stations. Soil moisture and ET were standardized for each reanalysis dataset to facilitate the ensemble, the comparison across regions and the trend analysis.

### Sensitivity to the definition of flash drought

A sensitivity test of flash drought frequency to the thresholds of soil moisture and precipitation was presented in the supporting information. In the case of precipitation, the corresponding composite of soil moisture percentile was greater than 40% in most regions of China (Fig. S1d), which was too high to meet the requirement of a drought event. On the other hand, if the soil moisture percentile was used as the identification of flash droughts (Fig. 1), the corresponding composite of precipitation also showed an excessively negative anomaly (Fig. S1c). This is consistent with previous findings that precipitation anomaly is no longer needed if the soil moisture is used for the identification^9^. We also conducted the sensitivity test by changing the soil depths and soil moisture percentile thresholds (Fig. S2). We found that the difference in the magnitude of flash drought frequency by using different soil depths was smaller than that by using different percentile thresholds. If the threshold of 20% or 30% was used, the flash drought frequency over northern and northwestern China was too low to have a statistical meaning within a 32-year period. And the consideration of a 2-m soil would cover both surface and root-zone, which facilitated the analysis of the impact of flash drought on vegetation. Therefore, we used the 2-m soil depth with a 40% threshold for the flash drought analysis. In addition, we tested the sensitivity of flash drought frequency to the thresholds of the surface air temperature and ET (Fig. S3). We found that if the thresholds of 1.5 or 2 standard deviations were used for temperature, or if 0.5 standard deviation was used for ET, the flash drought frequency over arid and semiarid regions was too low to have a statistical meaning for the whole period.

### Trend estimates and statistical significance test

Trends were calculated as the Sen median slope^38^, which was widely used in detecting monotonic trend in hydrometeorological time series^39^,^40^. The nonparametric Mann-Kendall test was performed for the statistical significance of trends. Note that we obtained similar results by using the linear regression and Student’s t-test in this paper.

### Contributions from component variables

The multiple linear regression was used to quantify the contributions of the surface air temperature (T), soil moisture (SM) and ET to the trend in flash droughts^41^,^42^. First, we obtained the regressed flash drought frequency based on a multiple regression analysis at each station:


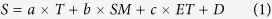


where *a*, *b* and *c* are the regression coefficients; and *D* is a constant; *S* is the flash drought frequency. Then, the contribution of the change in T to the change in flash drought (CT) was obtained as follows:


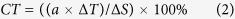


where Δ*S* and Δ*T* are the changes in mean *S* and T between the first and last five years. The contributions of the changes in SM and ET to the change in flash droughts could be calculated in the same manner. We should note that the estimates of contributions from component variables are based on a multiple linear regression by assuming that the component variables are independent from each other, while these components sometimes have interactions.

## Additional Information

**How to cite this article**: Wang, L. *et al.* Increasing flash droughts over China during the recent global warming hiatus. *Sci. Rep.* **6**, 30571; doi: 10.1038/srep30571 (2016).

## Supplementary Material

Supplementary Information
