# Supplementary material for: Increasing flash droughts over China during the recent global warming hiatus
Source: Sci Rep. 2016 Aug 11;6:30571. doi: 10.1038/srep30571 (PMC4980985; doi:10.1038/srep30571)
Supplement: Supplementary Information [file srep30571-s1.doc]

Supplementary Information to

**Increasing flash droughts over China during the recent global warming hiatus**

Linying Wang^1,2^, Xing Yuan1[[1]](#footnote-2)*, Zhenghui Xie^3^, Peili Wu^4^, Yaohui Li^5^

^1^RCE-TEA, Institute of Atmospheric Physics, Chinese Academy of Sciences, Beijing 100029, China

^2^University of Chinese Academy of Sciences, Beijing 100049, China

^3^LASG, Institute of Atmospheric Physics, Chinese Academy of Sciences, Beijing 100029, China

^4^Met Office Hadley Centre, Exeter EX1 3PB, UK

^5^Institute of Arid Meteorology, China Meteorological Administration, Lanzhou 730020, China

Submitted to *Scientific Reports*

28 May 2016

**Table S1.** Lists of p-values for Mann-Kendall trends of flash drought event and its component variables of temperature (T), soil moisture (SM) and ET averaged over China and the sub-regions for the period of 1979-2010. The positive and negative signs in the brackets indicate increasing and decreasing trends respectively.

| Region | Drought | Tave | SM | ET |
| --- | --- | --- | --- | --- |
| China | 1.72E-06(+) | 1.73E-05(+) | 1.71E-02(-) | 1.57E-02(+) |
| SC | 1.21E-04(+) | 9.30E-05(+) | 8.21E-03(-) | 8.13E-05(+) |
| NC | 2.32E-04(+) | 2.99E-04(+) | 5.70E-01(-) | 1.40E-01(+) |
| NEC | 2.32E-04(+) | 1.38E-04(+) | 4.61E-02(-) | 5.93E-01(+) |

**Table S2.** The same as Table S1, but for the sub-periods of 1979-1997 and 1998-2010.

| Period | Region | Drought | Tave | SM | ET |
| --- | --- | --- | --- | --- | --- |
| 1979-1997 | China | 0.093(+) | 0.108(+) | 0.944(-) | 0.401(+) |
|  | SC | 0.441(+) | 0.441(+) | 0.780(-) | 0.363(+) |
|  | NC | 0.162(+) | 0.108(+) | 0.624(-) | 0.363(+) |
|  | NEC | 0.780(-) | 0.108(+) | 0.263(+) | 0.484(+) |
| 1998-2010 | China | 0.077(+) | 0.760(-) | 0.161(-) | 0.200(+) |
|  | SC | 0.044(+) | 0.428(+) | 0.044(-) | 0.077(+) |
|  | NC | 0.855(+) | 0.127(-) | 0.428(+) | 0.360(+) |
|  | NEC | 0.300(+) | 0.360(-) | 0.059(-) | 0.951(-) |


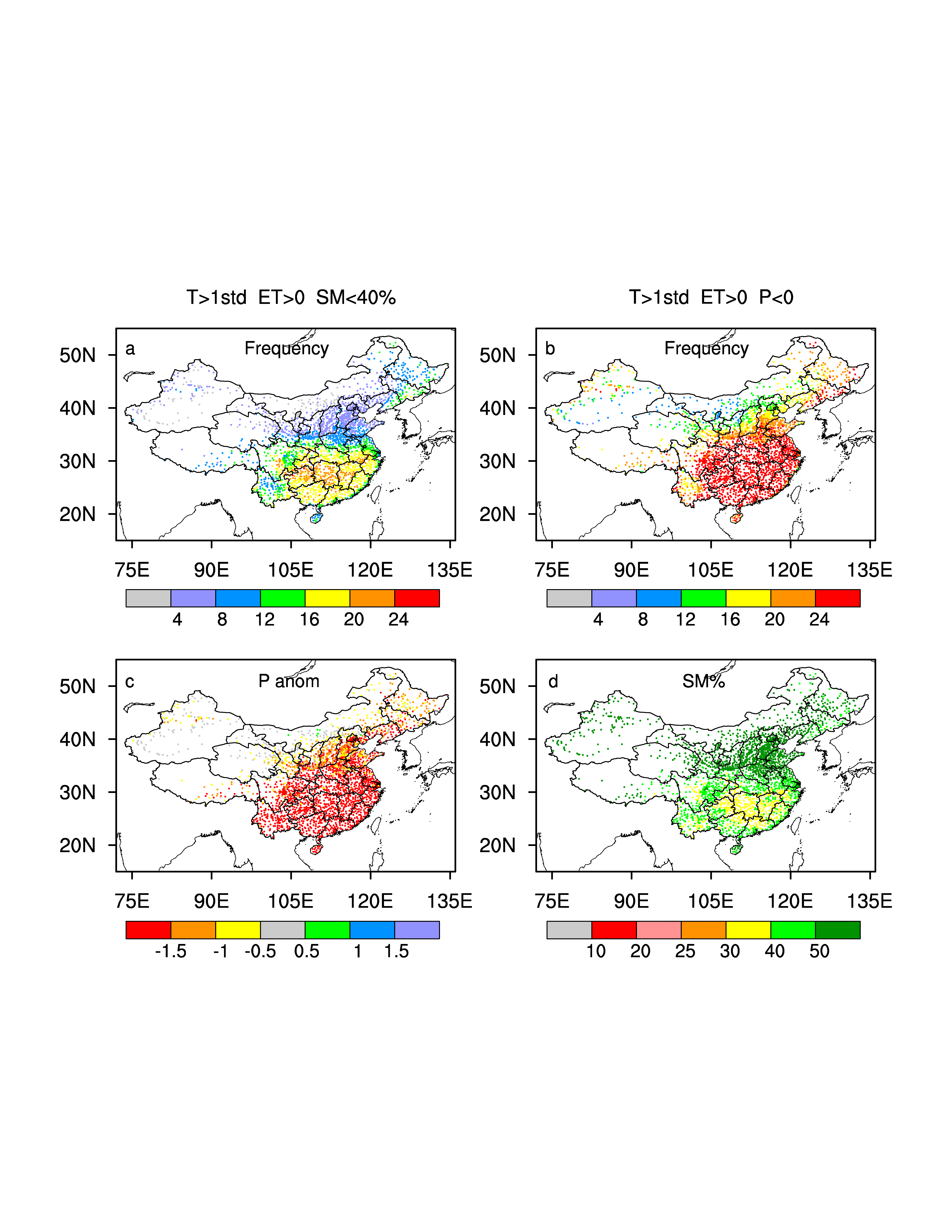


**Figure S1.** Sensitivity of the flash drought frequency to the thresholds of soil moisture (SM) and precipitation (P). (a) Ensemble mean frequency of flash drought events, where the flash droughts are defined the same as Figure 1. The unit is in events per decade. (b) As for a, the flash droughts are defined the same as a, but with P anomaly < 0 instead of SM% < 40%. (c) Ensemble mean composite of P anomalies for pentads under flash droughts defined in a, and the unit is mm/day. (d) Ensemble mean composite of SM% for pentads under flash droughts defined in b, and the unit is in percent. The ensemble mean is the average of the composite from each reanalysis product. The figure was created by the NCAR Command Language (Version 6.3.0) [Software]. (2016). Boulder, Colorado: UCAR/NCAR/CISL/TDD. http://dx.doi.org/10.5065/D6WD3XH5.


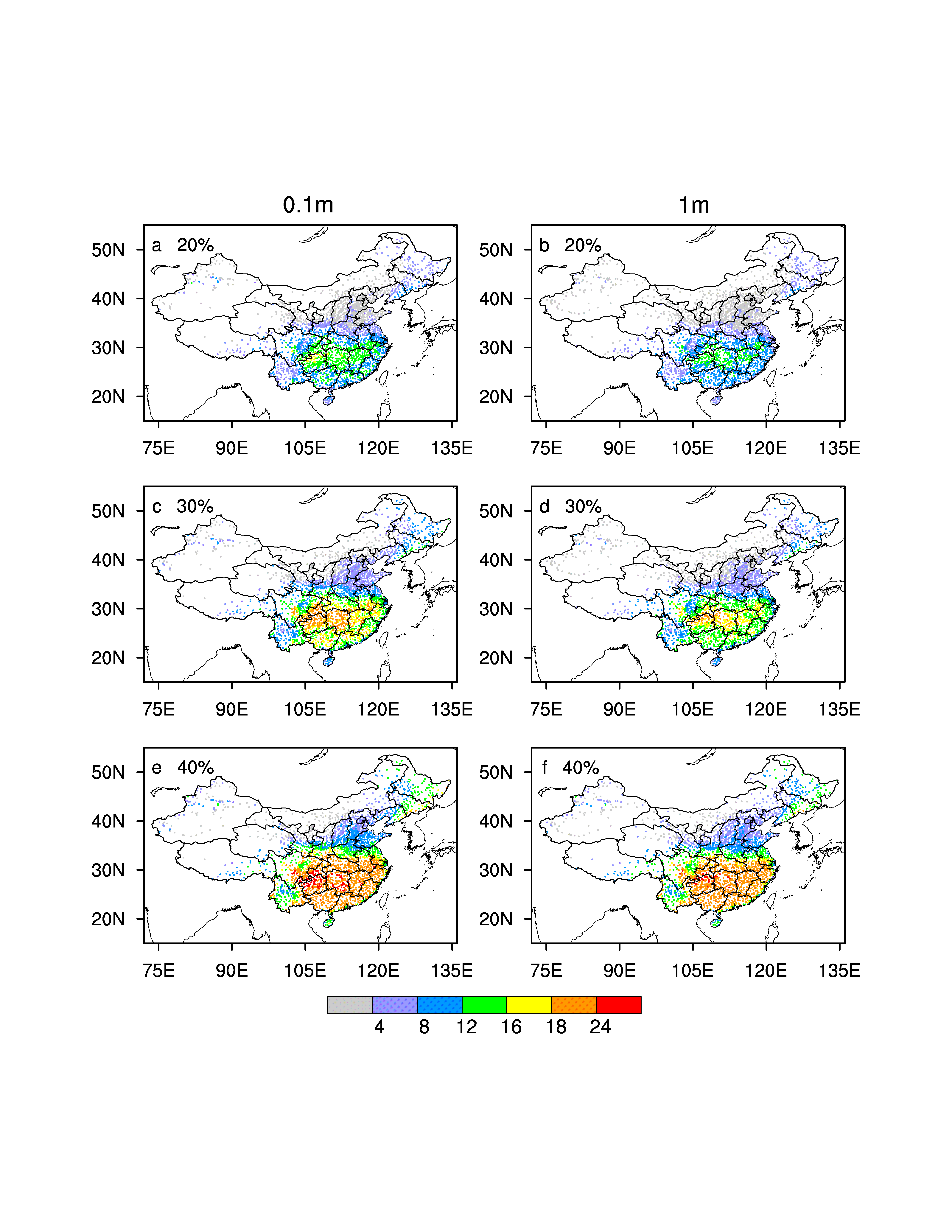


**Figure S2.** Sensitivity of the flash drought frequency to the soil depths and percentile thresholds used for the definition. (a) Ensemble mean frequency of flash drought events for SM% < 20%, SM depth is 0.1m. (b) As for a, but SM depth is 1m. (c)-(d) As for a and b, but SM% < 30%. (e)-(f) As for a and b, but SM% < 40%. The ensemble mean is the average of the frequency from each reanalysis product. The figure was created by the NCAR Command Language (Version 6.3.0) [Software]. (2016). Boulder, Colorado: UCAR/NCAR/CISL/TDD. http://dx.doi.org/10.5065/D6WD3XH5.


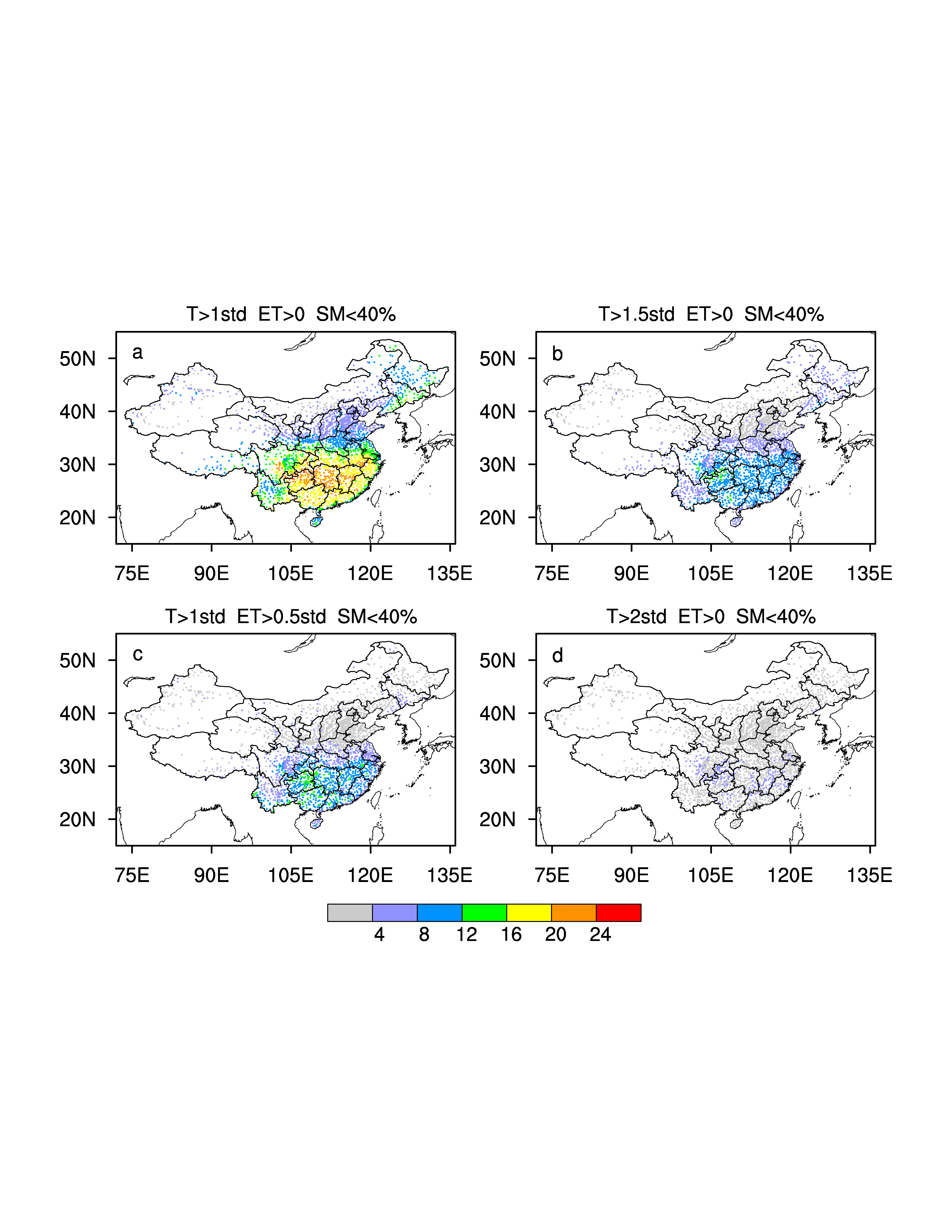


**Figure S3.** Sensitivity of the flash drought frequency to surface air temperature (T) and ET used for the definition. (a) Ensemble mean frequency of flash drought events for T anomaly > one standard deviation, ET anomaly > 0, and SM% < 40%. (b) As for a, but T anomaly > 1.5 standard deviation. (c) As for a, but ET anomaly > 0.5 standard deviation. (d) As for a, with T anomaly > 2 standard deviation. The ensemble mean is the average of the frequency from each reanalysis product. The figure was created by the NCAR Command Language (Version 6.3.0) [Software]. (2016). Boulder, Colorado: UCAR/NCAR/CISL/TDD. http://dx.doi.org/10.5065/D6WD3XH5.


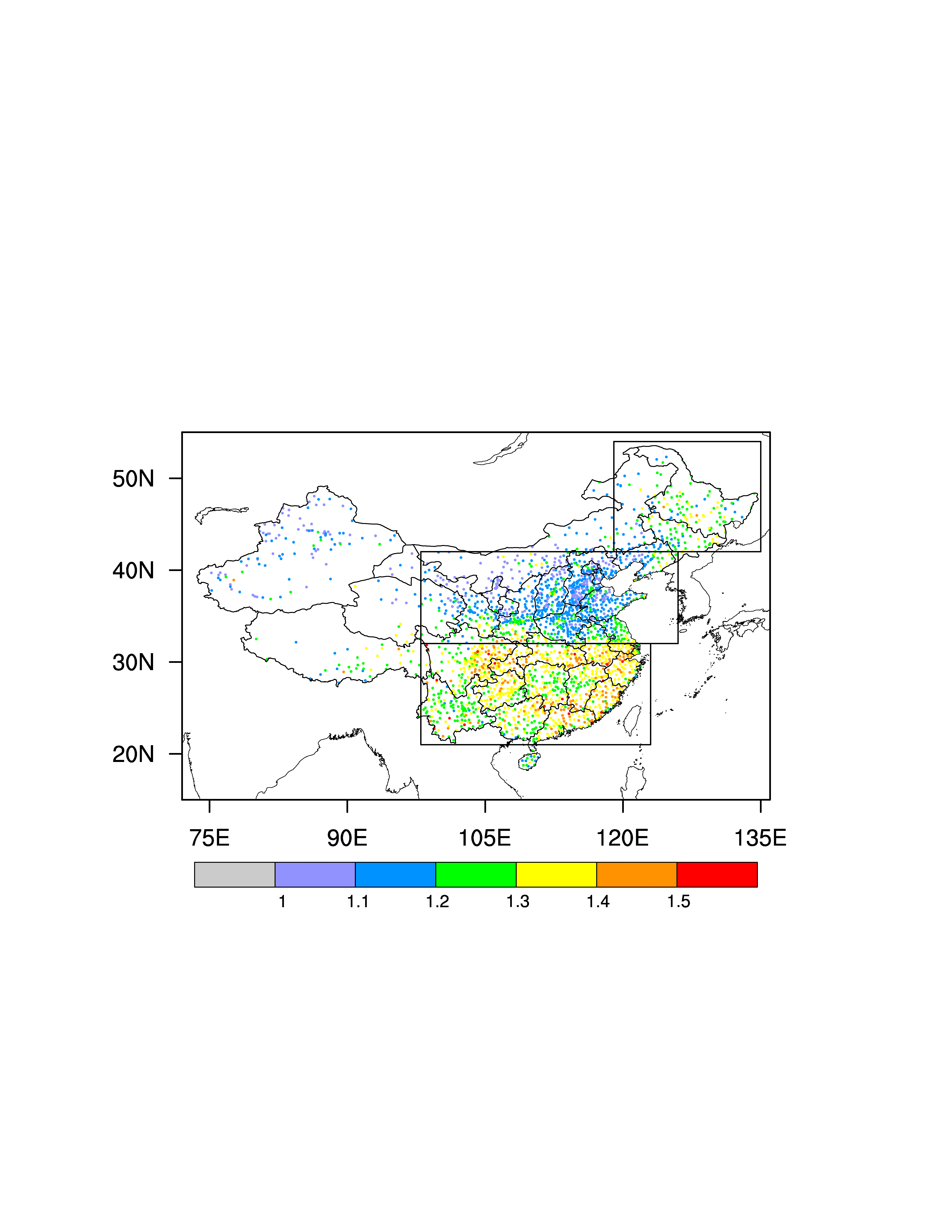


**Figure S4.** Ensemble mean duration of flash drought events. The flash droughts are defined the same as Figure 1. The mean duration is defined as the average number of pentads each flash drought event experience. The ensemble mean is the average of the duration from each reanalysis product. The unit is in pentad. The figure was created by the NCAR Command Language (Version 6.3.0) [Software]. (2016). Boulder, Colorado: UCAR/NCAR/CISL/TDD. http://dx.doi.org/10.5065/D6WD3XH5.

1. ^*^*Corresponding author address:* Xing Yuan, RCE-TEA, Institute of Atmospheric Physics, Chinese Academy of Sciences, Beijing 100029, China. E-mail: yuanxing@tea.ac.cn [↑](#footnote-ref-2)
